# Chloral and Camphor in the Treatment of Chancroid

**Published:** 1893-05

**Authors:** 


CHLORAL AND CAMPHOR IN THE TREATMENT OF CHANCROID. —In the March number of the *Annales des malacies des organes genito-urinaries* there is a summary of an article by Dr. E. Cavazzani, published in the *Giornale italiano delle malattie veneree e della pelle*, on the treatment of soft chancre with a mixture of five parts of chloral hydrate, three of camphor, and twenty-five of glycerine. The author reports twenty-six cases treated with this application in which a cure was attained in from two to eighteen days. It is said that the secretion diminishes rapidly and soon ceases altogether, that the local inflammation subsides notably, that the epithelium is regenerated speedily, and that suppurating buboes are a rarity.—*Ex.*

